# Deletion of Thioredoxin Interacting Protein (TXNIP) Augments Hyperoxia-Induced Vaso-Obliteration in a Mouse Model of Oxygen Induced-Retinopathy

**DOI:** 10.1371/journal.pone.0110388

**Published:** 2014-10-16

**Authors:** Mohammed A. Abdelsaid, Suraporn Matragoon, Adviye Ergul, Azza B. El-Remessy

**Affiliations:** 1 Clinical and Experimental Therapeutics, University of Georgia, Augusta, Georgia, United States of America; 2 Culver Vision Discovery Institute, Augusta, Georgia, United States of America; 3 Department of Physiology, Georgia Regents University, Augusta, Georgia, United States of America; 4 Charlie Norwood VA Medical Center, Augusta, Georgia, United States of America; Children's Hospital Boston, United States of America

## Abstract

We have recently shown that thioredoxin interacting protein (TXNIP) is required for VEGF-mediated VEGFR2 receptor activation and angiogenic signal. Retinas from TXNIP knockout mice (TKO) exhibited higher cellular antioxidant defense compared to wild type (WT). This study aimed to examine the impact of TXNIP deletion on hyperoxia-induced vaso-obliteration in ischemic retinopathy. TKO and WT pups were subjected to oxygen-induced retinopathy model. Retinal central capillary dropout was measured at p12. Retinal redox and nitrative state were assessed by reduced-glutathione (GSH), thioredoxin reductase activity and nitrotyrosine formation. Western blot and QT-PCR were used to assess VEGF, VEGFR-2, Akt, iNOS and eNOS, thioredoxin expression, ASK-1 activation and downstream cleaved caspase-3 and PARP in retinal lysates. Retinas from TKO mice exposed to hyperoxia showed significant increases (1.5-fold) in vaso-obliteration as indicated by central capillary drop out area compared to WT. Retinas from TKO showed minimal nitrotyrosine levels (10% of WT) with no change in eNOS or iNOS mRNA expression. There was no change in levels of VEGF or activation of VEGFR2 and its downstream Akt in retinas from TKO and WT. In comparison to WT, retinas from TKO showed significantly higher level of GSH and thioredoxin reductase activity in normoxia but comparable levels under hyperoxia. Exposure of TKO to hyperoxia significantly decreased the anti-apoptotic thioredoxin protein (∼50%) level compared with WT. This effect was associated with a significant increase in activation of the apoptotic ASK-1, PARP and caspase-3 pathway. Our results showed that despite comparable VEGF level and signal in TKO, exposure to hyperoxia significantly decreased Trx expression compared to WT. This effect resulted in liberation and activation of the apoptotic ASK-1 signal. These findings suggest that TXNIP is required for endothelial cell survival and homeostasis especially under stress conditions including hyperoxia.

## Introduction

Imbalance in cellular redox system has been linked to be several cardiovascular disorders such as ischemic heart disease, inflammation, atherosclerosis and aberrant angiogenesis (reviewed in [1]-[3]). The thioredoxin (Trx) system, major regulator of antioxidant defense represents an attractive target for oxidative stress-associated disorders. Trx is a multifunctional protein that acts as a protein disulfide reductase and participates in redox-dependent processes [3], including protein folding, regulation of apoptosis and antioxidant protection from oxidative stress. Trx has 2 isoforms, cytosolic/nuclear (Trx-1) and mitochondrial (Trx-2). Overexpression of Trx in transgenic mice increases the resistance to various oxidative stresses and attenuates ischemic damage in the brain [4] and myocardial infarction [5]. Expression and activity of Trx are tightly regulated by the endogenous inhibitor thioredoxin-interacting protein (TXNIP), where TXNIP binds Trx and limit its ability to interact with other proteins [6]. As such, TXNIP regulates Trx-dependent cellular redox state and increases reactive oxygen species and oxidative stress [7].

TXNIP is a stress sensor and its expression can be induced to a various number of exogenous and endogenous stimuli including inflammation, metabolic stress, changes in calcium levels, and also in response to changes in oxygen levels [8]-[13]. Increased levels of TXNIP bind more thioredoxin limiting its anti-apoptotic effects by releasing the apoptosis signal–regulating kinase l (ASK-1) from the inhibitory complex [14], [15]. We and others showed that TXNIP plays a pro-apoptotic role as it binds and inhibits Trx releasing free ASK-1 resulting in cell death [8], [13], [16]–[19]. Although TXNIP lacks specific pharmacological inhibitor, studies using calcium channel blockers, quercetin or T-resveratrol demonstrated neuro- and vascular protective effects that were associated with TXNIP inhibition [13], [17], [20]–[22]. Genetic deletion of TXNIP (TKO) demonstrated significant increases in antioxidant defense compared to wild-type mice [23], [24]. Retinas from TKO showed similar vascular density to WT littermates as recently characterized by our group [23]. Interestingly, TXNIP expression is required to achieve homeostasis of redox state in endothelial cells [23], [25]. Silencing TXNIP expression impaired VEGF receptor activation and angiogenic response VEGF via redox-dependent and independent pathways [23], [25]. Here we examined the impact of TXNIP deletion on hyperoxia-induced vaso-obliteration. Oxygen induced retinopathy model is a well-established model that utilize high oxygen to induce oxidative stress, endothelial cell ischemia and apoptosis in the developing retina [26]. Our initial hypothesis was that TKO mice will be protected against the hyperoxia induced vaso-obliteration. Instead, our results showed that TKO mice are more vulnerable to oxygen induced retinopathy model. The current study investigates the molecular mechanism involved to better understand the complex nature of redox regulation.

## Materials and Methods

### Animals

Experiments were approved by the Institutional Committee for Animal Use in Research and Education at Charlie Norwood VA medical Center (ACORP # 04-12-044) and conformed to the ARVO Statement for the Use of Animals in Ophthalmic and Vision Research. All experiments were performed using age-matched wild type (WT) mice C57Bl/6 mice (Jackson Laboratory, Bar Harbor, Maine) and TXNIP knockout mice (TKO) that was provided as a kind gift from Dr. AJ Lusis and Dr. ST Hui at the BioSciences Center, San Diego State University, San Diego, CA. TKO mice have a global knockout of the expression of functional TXNIP as characterized previously [24]. TKO mice are similar in weight and activity to WT or heterozygous littermates, with no differences in food consumption or litter sizes.

### TKO breeding and genotyping

Littermates of WT and homozygous TKO were used and genotyping was performed as described previously [24]. Briefly, DNA was prepared by incubating ear tissue with proteinase K and digestion buffer for one hour at 95°C. A mixture of primer sequence (5′-TGA-GGT-GGT-CTT-CAA-CGA-CC-3′. 5′GGA-AAG-ACA-ACG-CCA-GAA-GG-3′ and 5′-CCT-TGA-GGA-AGC-TCG-AAG-CC-3′ (IDT San Diego, CA)), buffer and 2 mM MgCl_2_ and polymerase enzyme (GoTAG Hot start polymerase, Promega, Madison, WI) were added to the DNA template. DNA segments were amplified using the Master plex-RealPlex2 (Eppendorf, Germany) and were detected with 1% agarose gel electrophoresis. Deleted TXNIP allele was detected at 530 bp while wild type was detected at 699 bp.

### Oxygen induced retinopathy model

Oxygen induced retinopathy was performed as described previously [27]. Briefly, on postnatal day 7 (p7), mice were placed along with their dam into a custom-built chamber (Biospherix, Redfield, NY) in which the partial pressure of oxygen was maintained at 70% for 5 days. At p12 pups were deeply anesthetized by IP injection of Avertin 240 mg/kg and sacrificed by jugular vein cut. One eye was enucleated and fixed in 2% paraformaldehyde overnight to be flat- mounted for vascular density. For the other eye, retinas were isolated and snap frozen for biochemical assays.

### Assessment of retinal vascular density and central capillary dropout areas

We examined the effect of TXNIP deletion on retinal vascular development at post-natal day (p7) in normoxic animals. Retinal capillary dropout areas were analyzed at p12 after hyperoxic exposure. Retinas of both wild type (WT) and TXNIP knockout (TKO) mice were inoculated and fixed in 4% paraformalhyde and flat-mounted. Retinas were labeled with the red fluorescent Alexa Fluor 594 isolectin GS-IB_4_ conjugate (Molecular Probes, Life Technology, Grand Island, NY) to quantify retinal vascular density. Retinas were viewed and imaged with fluorescence AxioObserver Zeiss Microscope (Germany). Images were then processed using Image J software to skeletonize and quantify the vascular density as described previously [23], [27]. Results were expressed as percentage of the total retinal area.

### Oxidized- and reduced-glutathione

Reduced glutathione was measured using the Northwest Life Science kit (Vancouver, WA) as described before [23], [27]. Briefly, reduced-GSH was calculated by subtracting the oxidized-GSSG from the total glutathione. For total glutathione, cells were lysed in phosphate buffer (100 mM potassium phosphate and 1 mM EDTA) and were mixed with an equal volume of 10 mM 5, 5′-dithiobis 2-nitrobenzoic acid (DTNB) in the presence of glutathione reductase and NADPH producing a yellow color measured at 412 nm. To detect GSSG, samples were treated with 10 mM 2-vinylpyridine (Sigma) in ethanol to sequester all the reduced GSH then measured using the same protocol as the total glutathione.

### Thioredoxin reductase activity (Trx-R)

Thioredoxin reductase activity was performed using a kit (Sigma) as described previously [8], [17]. Briefly, retinal samples were homogenized in assay buffer followed by the addition of DTNB with NADPH. Reduction of DTNB produced a strong yellow color that was measured at 412 nm. Thioredoxin reductase activity was measured as the difference between DTNB-reaction measurement of each sample in the presence and absence of a selective thioredoxin reductase inhibitor (provided in the kit) and expressed as unit/µg/min.

### Quantitative real time PCR

The One-Step qRT-PCR kit (Invitrogen) was used to amplify 10 ng retinal mRNA and quantification was performed as described previously [23]. PCR primers (listed in Table 1) were purchased from Integrated DNA Technologies Inc. (IDT, Coralville, IA). Quantitative PCR was performed using a Realplex Master cycler (Eppendorf, Germany). Expression of TXNIP, Trx-1, Trx-2, VEGF, eNOS, iNOS was normalized to the 18S level and expressed as relative expression to control.

**Table 1:** Primer sequence used to quantify mRNA expression levels using PCR analysis.

Gene	Forward primers	Reverse primers
TXNIP	5′AAGCTGTCCTCAGTCAGAGGCAAT3′	5′ATGACTTTCTTGGAGCCAGGGACA3′
VEGF	5′TGAGCCTTGTTCAGAGCGGAGAAA3′	5′TTCGTTTAACTCAAGCTGCCTCGC3′
Trx-1	5′TCAAGCCCTTCTTCCATTCC3′	5′GTCGGCATGCATTTGACTTC 3′
Trx-2	5′ CGCGGCTAGAGAAGATGGTC3′	5′TTGATGGCTAGCACGGTAGG3′
eNOS	5′ GCAGTGAAGATCTCTGCCTCA3′	5′AGAATGGTTGCCTTCACACG3′
iNOS	5′ CACCTTGGAGTTCACCCAGT3′	5′ ACCACTCGTACTTGGGATGC3′
18S	5′CGCGGTTCTATTTTGTTGGT3′	5′AGTCGGCATCGTTTATGGTC3′

### Western blot analysis

Protein expression in retina lysate was analyzed as described previously [23]. For VEGF, retinal lysates were subjected to heparin beads (Sigma) as described before [27], [28]. The beads were pelleted at 5000 × g for 1 min, washed in 400 mM NaCl and 20 mM Tris and loaded onto a 4-20% gradient Trisglycine pre-cast gel (BioRad). The primary antibodies were purchased as follow: VEGF (Rabbit polyclonal, EMD-Millipore), phosphor-VEGFR2, VEGFR2, phospho-Akt, Akt, phospho-ASK-1, ASK-1, cleaved caspase-3 (Rabbit polyclonal, Cell Signaling Tech, Danvers, MA), total Trx (Mouse monoclonal, Santa Cruz, Dallas, TX), and TXNIP (Rabbit polyclonal, Invitrogen, Grand Island, NY), cleaved PARP (BD Bioscience Pharmingen, San Diego, CA). Primary antibodies were detected using a horseradish peroxidase-conjugated antibody and enhanced chemiluminescence (GE Healthcare, Piscataway NJ).The films were scanned, and band intensity was quantified using densitometry software (Alpha Innotech Fluorchem, Santa Clara, CA).

### Detection of nitrotyrosine

Relative amounts of proteins nitrated on tyrosine were measured by use of slot-blot techniques as described previously [27]. In brief, retinal homogenate was immobilized onto nitrocellulose membrane by use of a slot-blot microfiltration unit (Bio-Rad Laboratories). After being blocked, membranes were incubated with a 1:500 dilution of nitrotyrosine (Calbiochem, San Diego, CA, USA) antibody followed by HRP-conjugated sheep anti-rabbit antibody and enhanced chemiluminescence (GE Healthcare). Relative levels of nitrotyrosine immunoreactivity were determined by densitometry software (Alpa Innotech).

### Data analysis

All the results were expressed as mean ± SE and the data were evaluated for normality and appropriate transformations were used when necessary. Vaso-obliteration was evaluated by analysis of variance, and the significance of difference between groups was assessed by the post-hoc test (Fisher's PLSD) and significance was defined as P <0.05. A two-way ANOVA was used to examine the effect of manipulation gene (WT vs. TKO) and oxygen levels (normoxia vs. hyperoxia) and their interaction on mRNA and expression of VEGF, TRX-1, TRX-2, eNOS and iNOS; expression of total TRX, NY, pAkt, pVEGFR2, pASK-1, Caspase-3 and PARP; TrxR activity and reduced GSH levels. Two-way ANOVA followed by Bonferroni post-tests were used to adjust for the multiple comparisons used to assess significant effects. NCSS 2007 was used for all analyses (NCSS, version 07.1.14 LLC, Kaysville, UT). Statistical significance was determined at alpha = 0.05.

## Results

### Deletion of TXNIP augments hyperoxia-induced retinal vaso-obliteration

**Figure 1:**
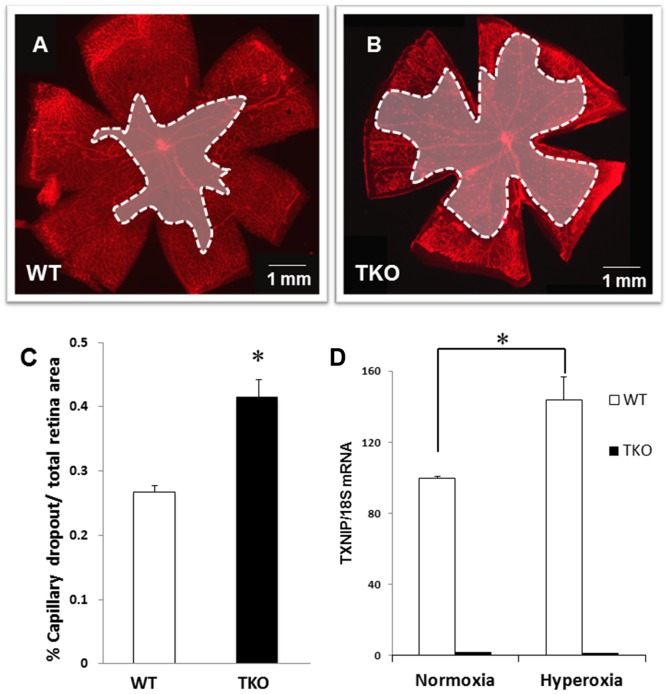
Deletion of TXNIP augments hyperoxia-induced vaso-obliteration compared to WT. Wild type (WT) and TXNIP knockout (TKO) mice were subjected to hyperoxia (75% O2, p7–p12). Retinas were fixed and stained with iso-lectin B4 to quantify oxygen induced vaso-obliteration. **A**–**C**) Retinas from TKO mice exposed to hyperoxia showed significant increases in vaso-obliteration compared to WT. (*P<0.05 vs WT, n = 12). **D**) Hyperoxia stimulates TXNIP expression mRNA in WT but not in TKO mice. (*P<0.05 vs WT normoxia, n = 4)

Exposure of the developing rodent retina (post-natal day p7 to p12) to high levels of oxygen causes vascular cell death as indicated by central capillary dropout [26]. Increases in oxidative stress and peroxynitrite have been shown to cause vascular cell loss in ischemic retinopathy model [27], [29]–[31]. Retinas from TKO demonstrate comparable vascular density to their WT littermates at normal oxygen level at p7 (Fig. S1) and at p12 as recently characterized by our group [23]. We and others have previously showed that deletion of TXNIP enhanced antioxidant defense and decreased oxidative stress [8], [23], [24]. Therefore, TKO mice were predicted to show higher vascular protection against hyperoxia compared to wild type (WT) mice. In contrast, deletion of TXNIP aggravated hyperoxia-induced vaso-obliteration as indicated by significant increase (1.6-fold) in central capillary dropout areas compared to WT at p12 (Fig. 1A–C). Of note, hyperoxia drives retinal TXNIP mRNA expression (1.5-fold) in WT mice but not TKO (Fig. 1D).

### Deletion of TXNIP decreases nitrative stress under normoxia and hyperoxia

**Figure 2:**
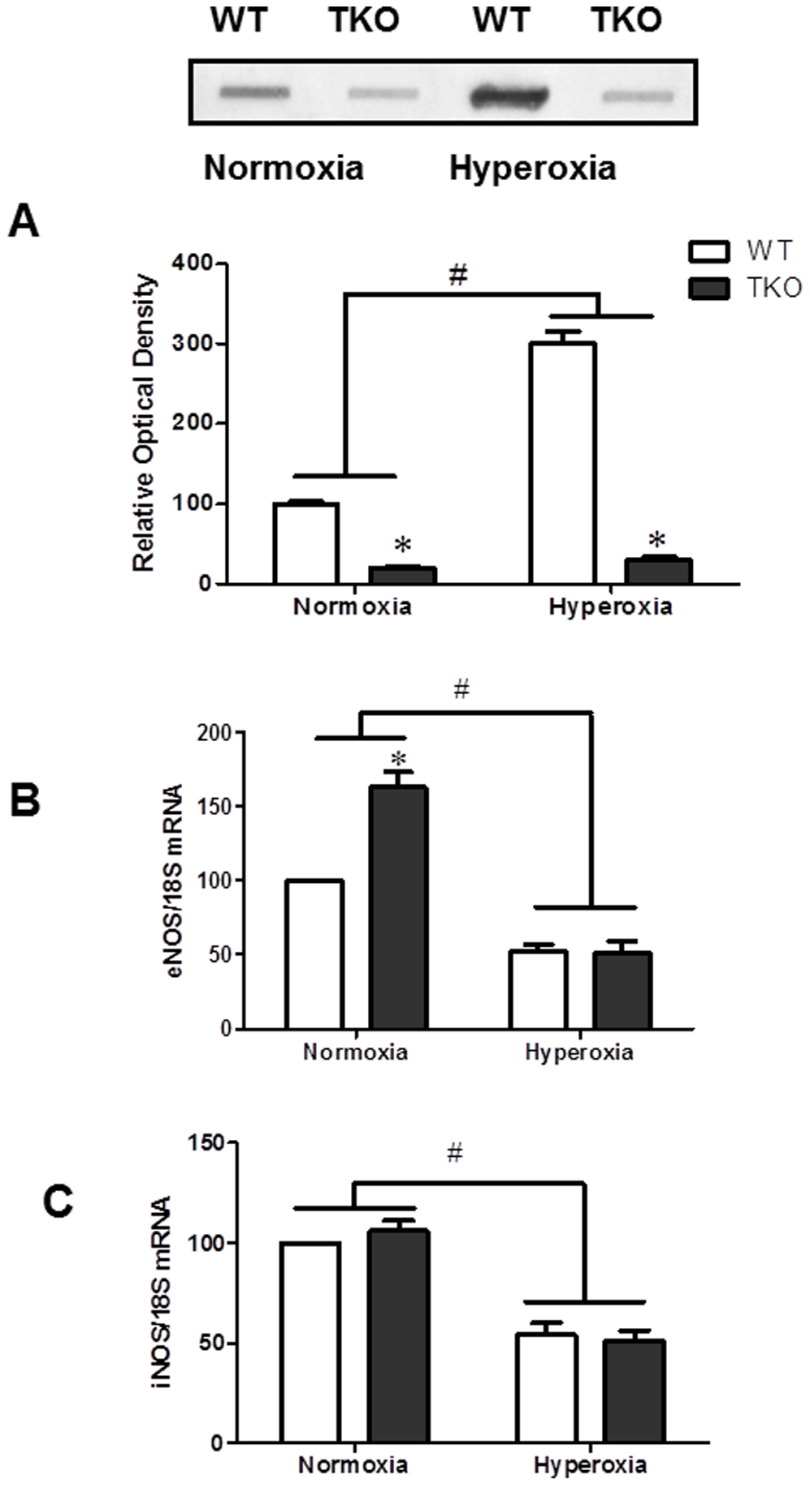
Deletion of TXNIP decreases nitrative stress under normoxia and hyperoxia. **A**) Retinas of TKO showed significantly less nitrotyrosine levels at normoxia or hyperoxia compared to WT. **B**) TKO showed higher eNOS mRNA level in normoxia. Hyperoxia significantly reduced eNOS mRNA levels. A 2×2 analysis showed a significant interaction between the gene (TKO) and the oxygen level (hyperoxia) in both nitrotyrosine and eNOS levels. C) We did not detect difference between TKO and WT in the expression of iNOS. Hyperoxia caused significant reduction of iNOS compared to normoxia. (#P<0.05 Hyperoxia vs Normoxia, *P<0.05, TKO vs WT, n = 4–6).

We next examined the levels of nitrotyrosine (NY), the footprint of peroxynitrite which is believed to mediate the detrimental effects of hyperoxia. As shown in Fig. 2A, hyperoxia significantly increased retinal NY formation (3-fold) in WT compared to normoxia. TKO showed minimal level of NY (10%) at normoxia and 20% at hyperoxia compared to WT. The two-way ANOVA showed a significant interaction between TKO and WT in response to high levels of oxygen (Fig. 2A). We also examined the expression of eNOS and iNOS at the mRNA level. Our results showed a significant interaction between TKO and WT in eNOS mRNA levels. TKO retinas showed 1.6-fold increase under normoxic conditions compared to WT-normoxia. Hyperoxia induced significant reduction in retinas from WT and TKO 52% and 51%, respectively compared to retinas from WT-normoxia (Fig. 2B). There was no significant interaction between TKO and WT in iNOS mRNA levels. Hyperoxia caused significant decrease in iNOS mRNA levels in both WT and TKO 54% and 51%, respectively compared to normoxia (Fig. 2C). These results suggest that TKO retinas had less tyrosine nitration at both normoxia and hyperoxia compared to WT.

### Deletion of TXNIP does not alter VEGF levels under normoxia and hyperoxia

**Figure 3:**
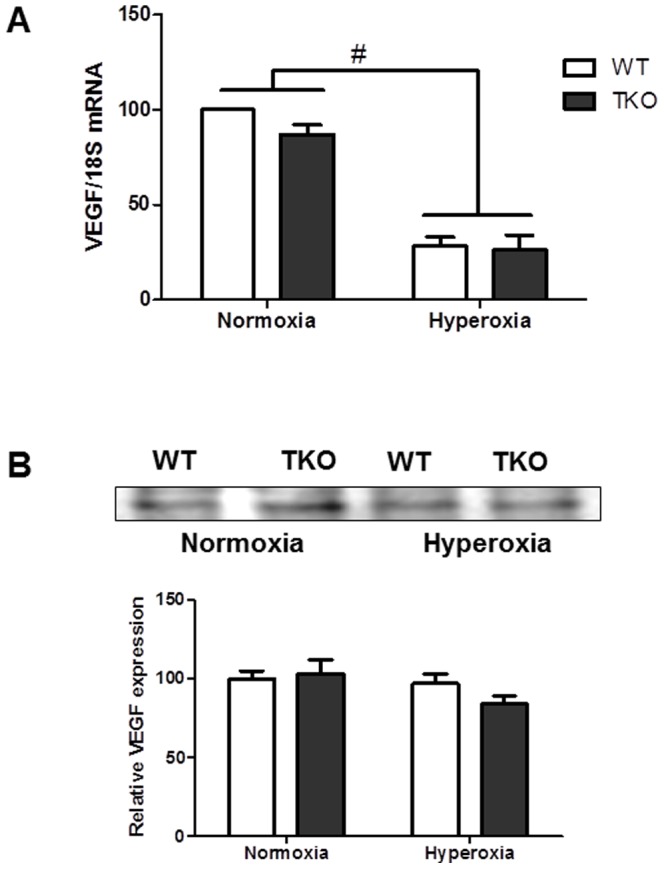
Deletion of TXNIP does not alter VEGF levels under normoxia or hyperoxia. (A) VEGF mRNA levels were detected from various groups using rt-PCR. (B) VEGF protein expression was examined using heparin-bound beads from p12 WT and TKO retinas. There was no change in levels of VEGF mRNA or VEGF expression between WT and TKO under normoxia. Hyperoxia caused significant decrease in VEGF mRNA compared to normoxia. Hyperoxia did not alter VEGF protein levels from normoxia in WT and TKO. (#P<0.05 Hyperoxia vs Normoxia, n = 4–6).

We next examined the effect of TXNIP deletion on VEGF levels under hyperoxia. Our 2×2 analysis showed no significant interaction between TKO and WT in the VEGF mRNA levels. We detected a significant decrease in the VEGF mRNA in groups exposed to hyperoxic conditions compared to the normoxia (Fig. 3A). On the other hand, we did not detect any significance interaction in the VEGF protein expression between groups (Fig. 3B). Our results show that TXNIP deletion did not alter VEGF protein levels compared to WT under normoxic or hyperoxic conditions.

### Deletion of TXNIP impairs VEGFR2/Akt activation in normoxia but not under hyperoxia

**Figure 4:**
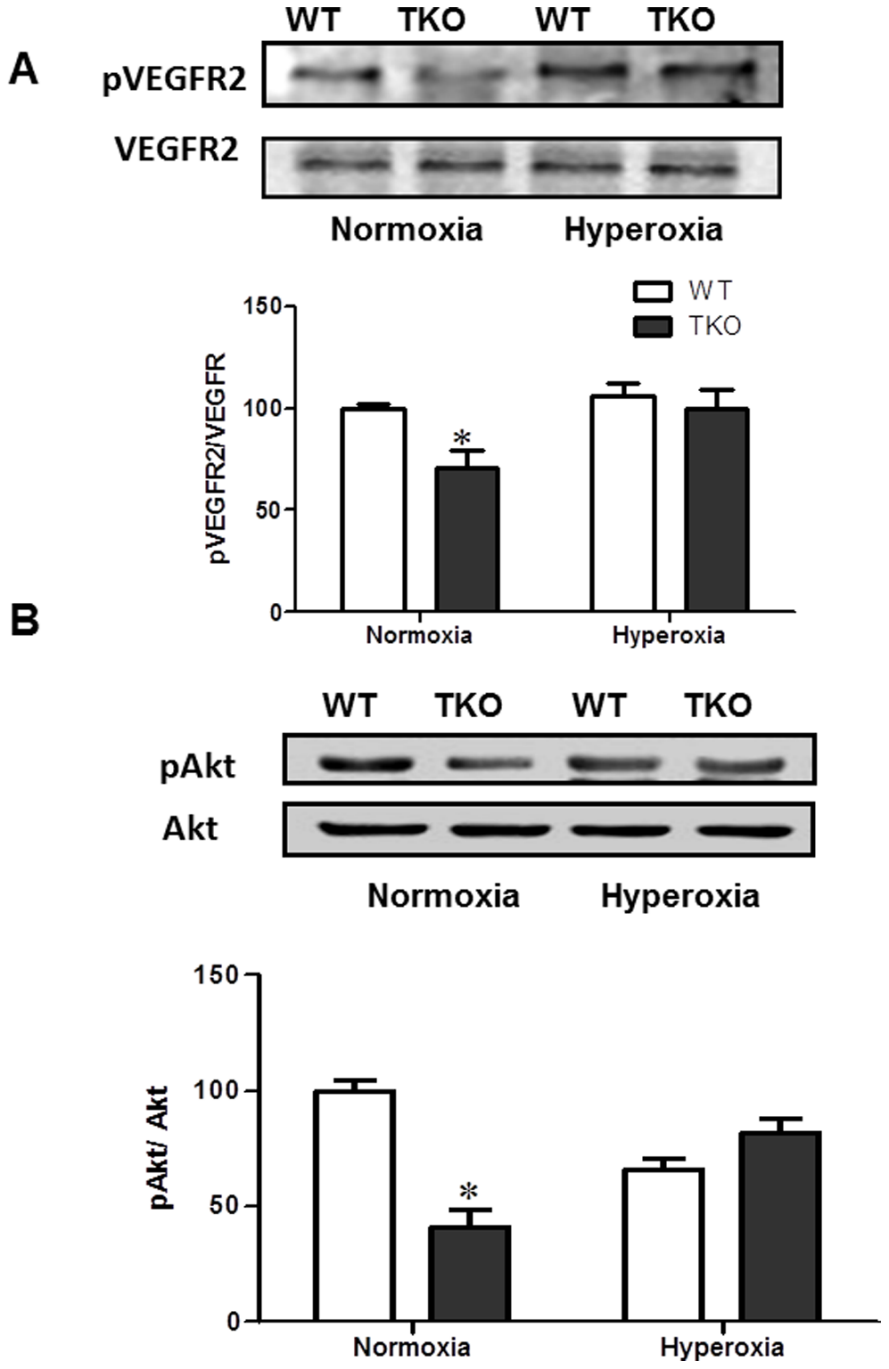
Deletion of TXNIP does not alter VEGFR2/Akt activation under hyperoxia. Wild type (WT) and TXNIP knockout (TKO) mice were subjected to hyperoxia (75% O2, p7–p12). Activation of VEGFR2 (**A**) and Akt (**B**) were examined as downstream signal of VEGF in p12 WT and TKO retinas. TKO showed significant decrease in phosphorylation of VEGFR-2 and Akt compared to WT under normoxic condition. We did not detect significant change in the activation of VEGFR2 and its downstream Akt in retinas from TKO and WT in response to hyperoxia. (*P<0.05, TKO vs WT, n = 4–6).

Our recent studies demonstrated that retinas from TKO mice showed similar level of VEGF but less VEGFR2 activation compared to WT under hypoxic condition [23]. Indeed, we detected a significant reduction in VEGFR2 phosphorylation in normoxic TKO retinas compared to normoxic WT (Fig. 4A). This effect was paralleled with significant decreases in the activation of survival protein Akt (Fig. 4B). Similar to our previous findings [27], hyperoxia decreased activation of Akt in WT compared to normoxic controls at p12 (Fig. 4B). Hyperoxia increased activation of VEGFR2 and its downstream target Akt in TKO mice to a comparable level with control WT. Two-way ANOVA did not show any significant interaction between TKO and WT in activation of VEGFR2 or Akt under hyperoxic conditions. Together, these results exclude the possibility that alteration in VEGF or VEGFR2 activation causes the aggravated vaso-obliteration response of TKO to hyperoxia.

### Deletion of TXNIP increases antioxidant defense and thioredoxin reductase activity

**Figure 5:**
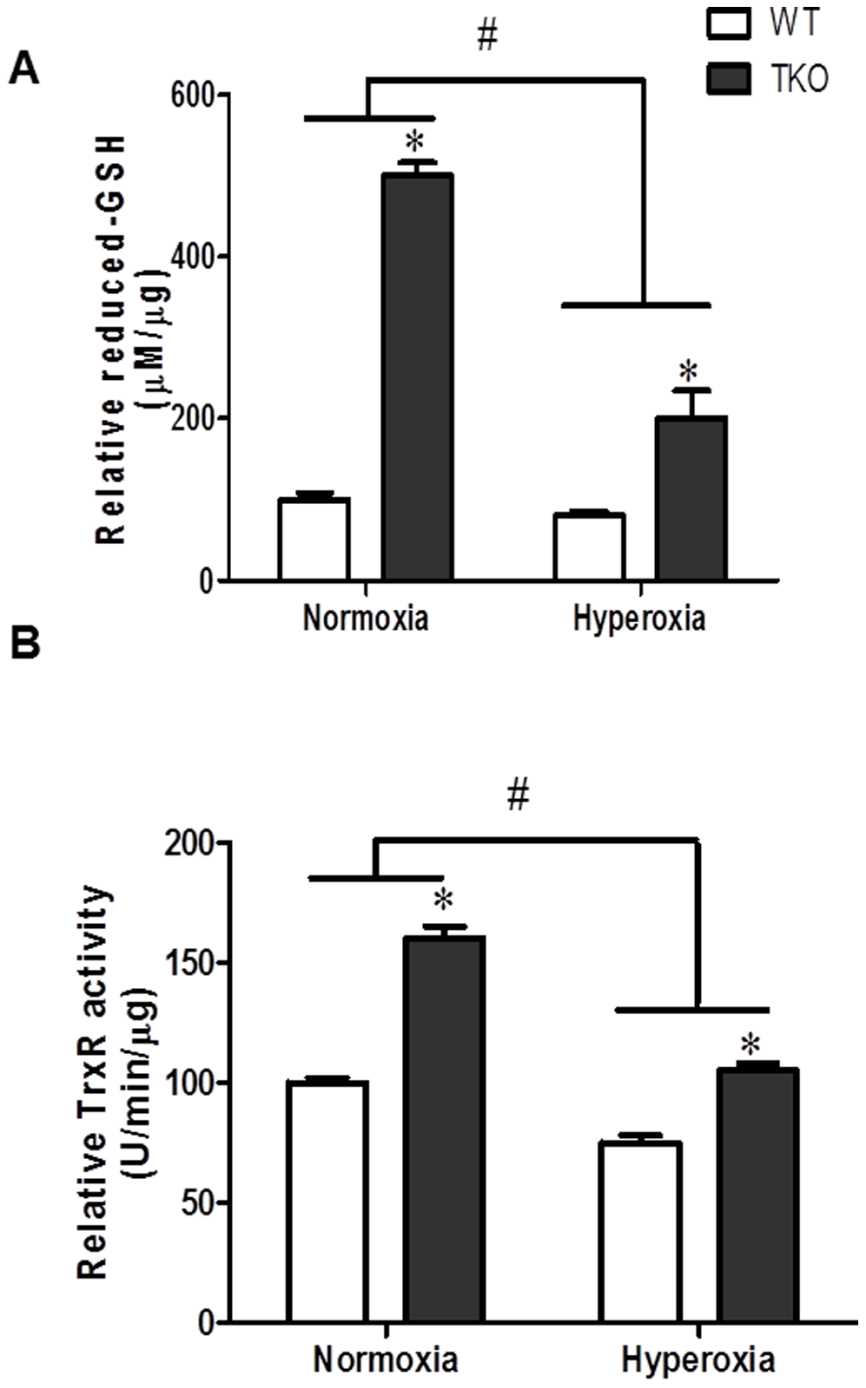
Deletion of TXNIP increases antioxidant defense level under normoxia and hyperoxia. In comparison to WT, retinas from TKO showed significantly higher level of reduced-GSH (**A**) and thioredoxin reductase activity (**B**) under normoxia. A 2×2 analysis showed a significant interaction between gene (TKO) and oxygen levels (hyperoxia) in both reduced-GSH and thioredoxin reductase activity measurements. (#P<0.05 Hyperoxia vs Normoxia, *P<0.05, TKO vs WT, n = 6–8).

We next examined reduced-glutathione levels (GSH) as marker of retinal antioxidant defense. As shown in Fig. 5A, TKO showed a significant interaction when compared to WT in the levels of GSH under normoxic (5-fold) and hyperoxic (2.25-fold) conditions. Similar trend was observed when we measured thioredoxin reductase (Trx-R) activity (Fig. 5B). Our results showed a significant interaction between TKO and WT in Trx-R activity when exposed to hyperoxia. TKO showed a significant increase in TrxR activity both under normoxic (1.6-fold) and hyperoxic conditions (1.25-fold) when compared to WT. These results confirmed that retinas from TKO had a higher antioxidant defense compared to WT.

### Deletion of TXNIP decreases Trx mRNA and protein levels under hyperoxia

**Figure 6:**
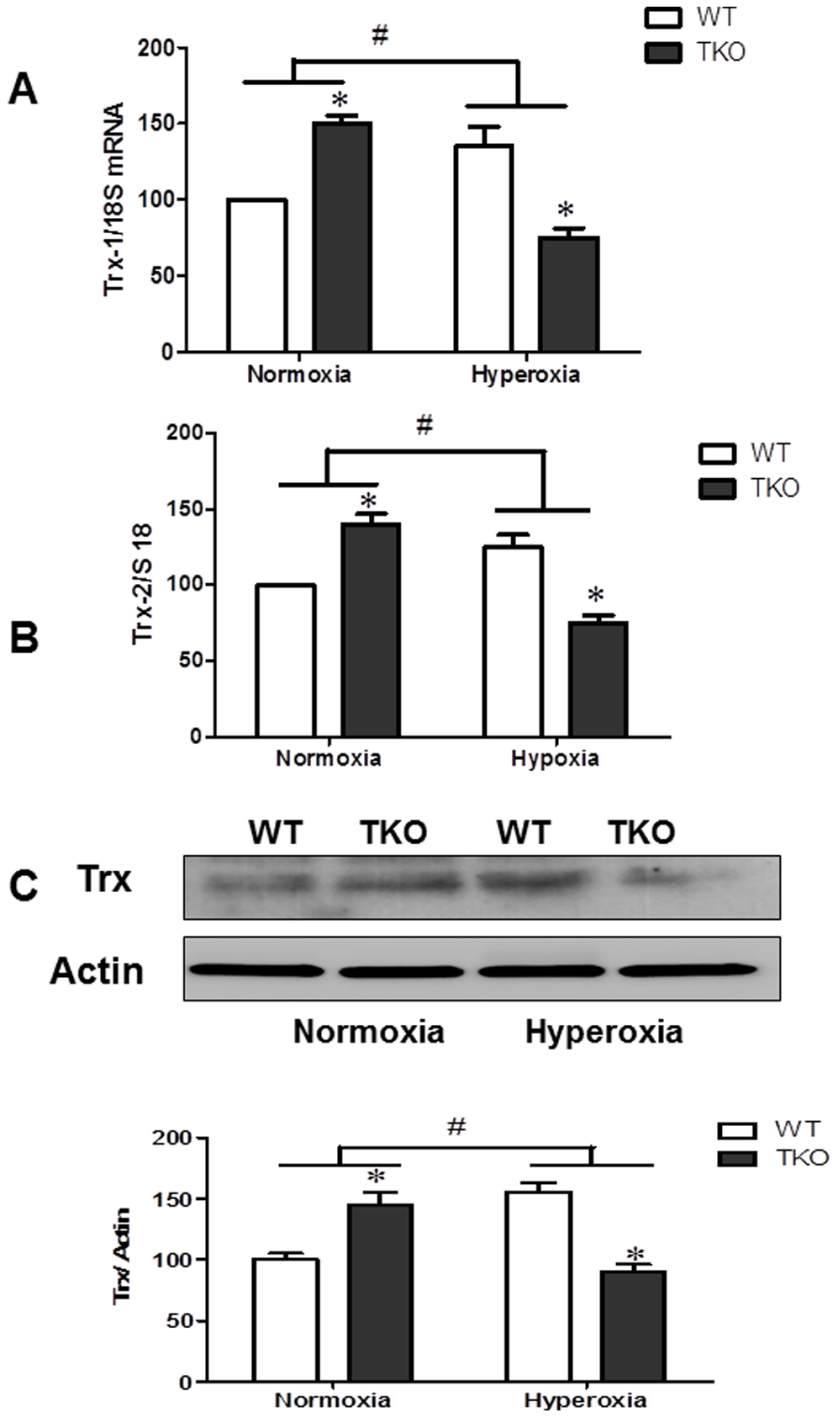
Deletion of TXNIP decreases thioredoxin levels under hyperoxic conditions. WT and TKO mice were subjected to 5 days hyperoxia (p7–12). Retinas were collected for protein and mRNA levels of thioredoxin (cytoplasmic Trx-1) (**A**) as well as mitochondrial Trx-2 (**B**) and total thioredoxin protein (**C**). A 2×2 analysis showed a significant interaction between gene (TKO) and oxygen levels (Hyperoxia) in both Trx-1 and Trx-2 as well as Trx total expression. Exposure of TKO pups to hyperoxia significantly decreased the anti-apoptotic thioredoxin on both mRNA as well as protein level compared to WT. (#P<0.05 Hyperoxia vs Normoxia, *P<0.05, TKO vs WT, n = 4–6).

As the Trx-R activity increases, the availability of free thioredoxin increases. We examined the effect of TXNIP deletion on levels of Trx levels in response to hyperoxia both on the transcriptional and expression levels. Trx has 2 isoforms, cytosolic/nuclear (Trx-1) and mitochondrial (Trx-2). Our results showed a significant interaction in the levels of Trx-1 and Trx-2 mRNA between gene (TKO and WT) under different oxygen level (normoxia vs hyperoxia). At normoxia, retinas from TKO had significantly higher mRNA expression of Trx-1 (1.5-fold) and Trx-2 (1.4-fold), respectively compared to WT (Fig. 6A–B). Hyperoxia induced mRNA levels 1.5-fold and 1.25 fold in Trx-1 and Trx-2, respectively in WT (Fig. 6A–B). In contrast, hyperoxia significantly decreased mRNA levels for Trx-1 (50%) and Trx-2 (45%) compared to TKO under normoxia (Fig. 6A–B). Next we examined total Trx protein expression and 2×2 analysis showed a significant interaction between TKO and WT in Trx protein expression. While total Trx expression was increased in TKO under normoxia (1.52 fold), hyperoxia caused a significant increase (1.5-Fold) in WT but a 40% reduction in the total Trx expression in TKO mice (Fig. 6C).

### TXNIP deletion increases ASK-1 activation and pro-apoptotic signal under hyperoxia

**Figure 7:**
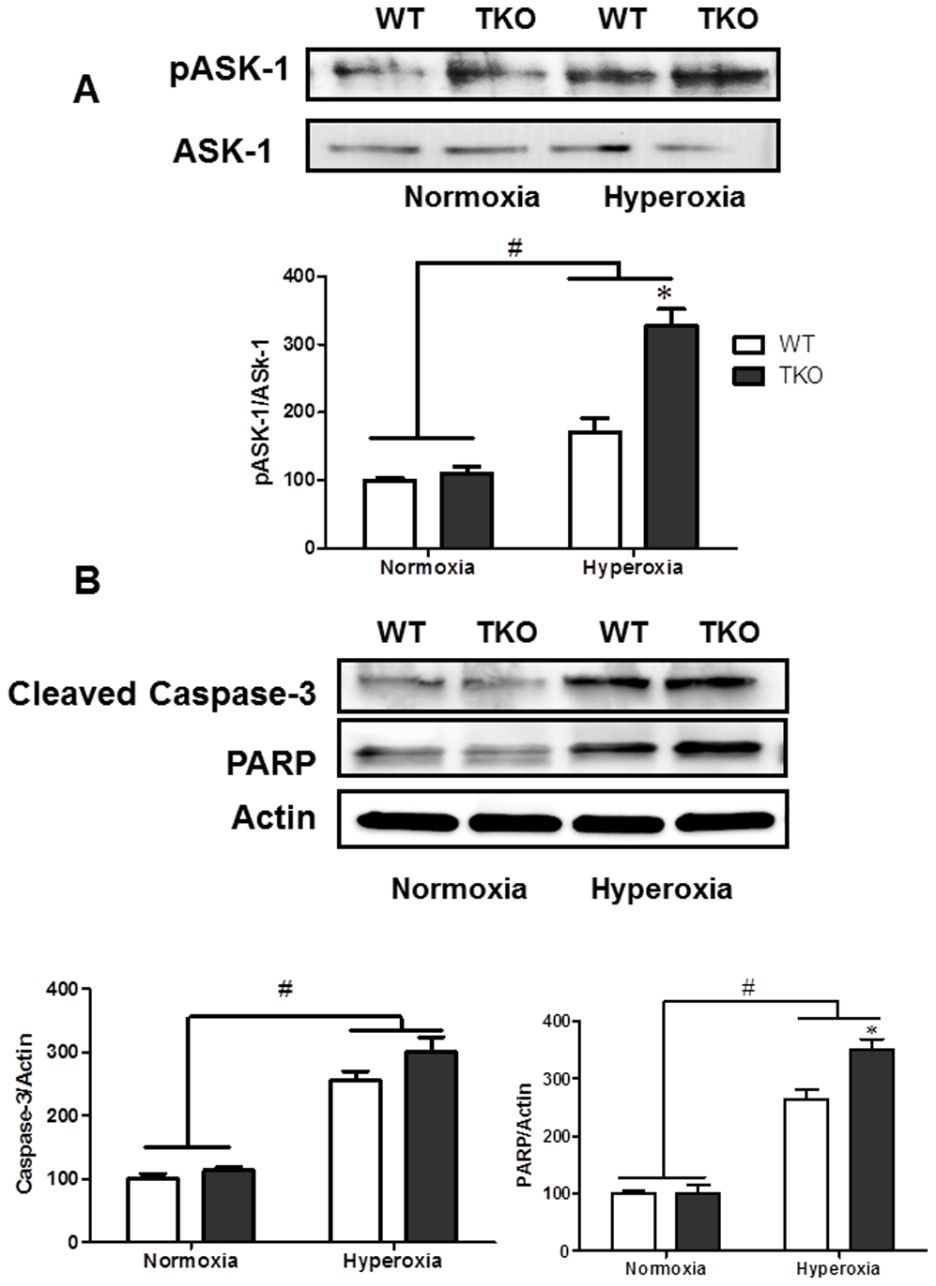
Deletion of TXNIP augments hyperoxia-induced ASK-1 activation and apoptotic markers. Wild type (WT) and TXNIP knockout (TKO) mice were subjected to 5 days hyperoxia (p7–12). Retinas were collected for protein ASK-1 (**A**) and apoptotic markers (Cleaved Caspase-3 and PARP) (**B**). A 2×2 analysis showed a significant interaction between gene (TKO) and oxygen levels (Hyperoxia) in activation of ASK-1. In parallen, Hyperoxia caused significant increase in cleaved caspase-3 and PARP expression compared to normoxia in WT and TKO. Reduced Trx levels were associated with a significant increase in activation of the apoptotic ASK-1, PARP and caspase-3 pathway. (#P<0.05 Hyperoxia vs Normoxia, *P<0.05, TKO vs WT, n = 4–6).

Trx is a negative regulator of the Apoptosis signal-regulating kinase-1 (ASK-1) pro-apoptotic pathway through direct binding to the N-terminal region of ASK-1 [32]. Hyperoxia decreased Trx expression in TKO animal, therefore, we examined the activation of ASK-1 and downstream apoptotic signal. A 2×2 analysis showed a significant interaction between TKO and WT in ASK-1 activation. While no significant difference was detected at normoxia, hyperoxia caused a significant increase in ASK-1 phosphorylation in TKO compared to WT (Fig. 7A). We next examined the downstream apoptotic signal in TKO mice. Although no significant interaction was detected between TKO and WT, hyperoxia caused increased apoptotic signal in both WT and TKO mice. In WT, hyperoxia caused 2.5-fold and 2.65-fold increase in caspase-3 cleavage and PARP expression respectively. In TKO, hyperoxia caused 3-fold and 3.5-fold increase in caspase-3 cleavage and PARP expression respectively.

## Discussion

**Figure 8:**
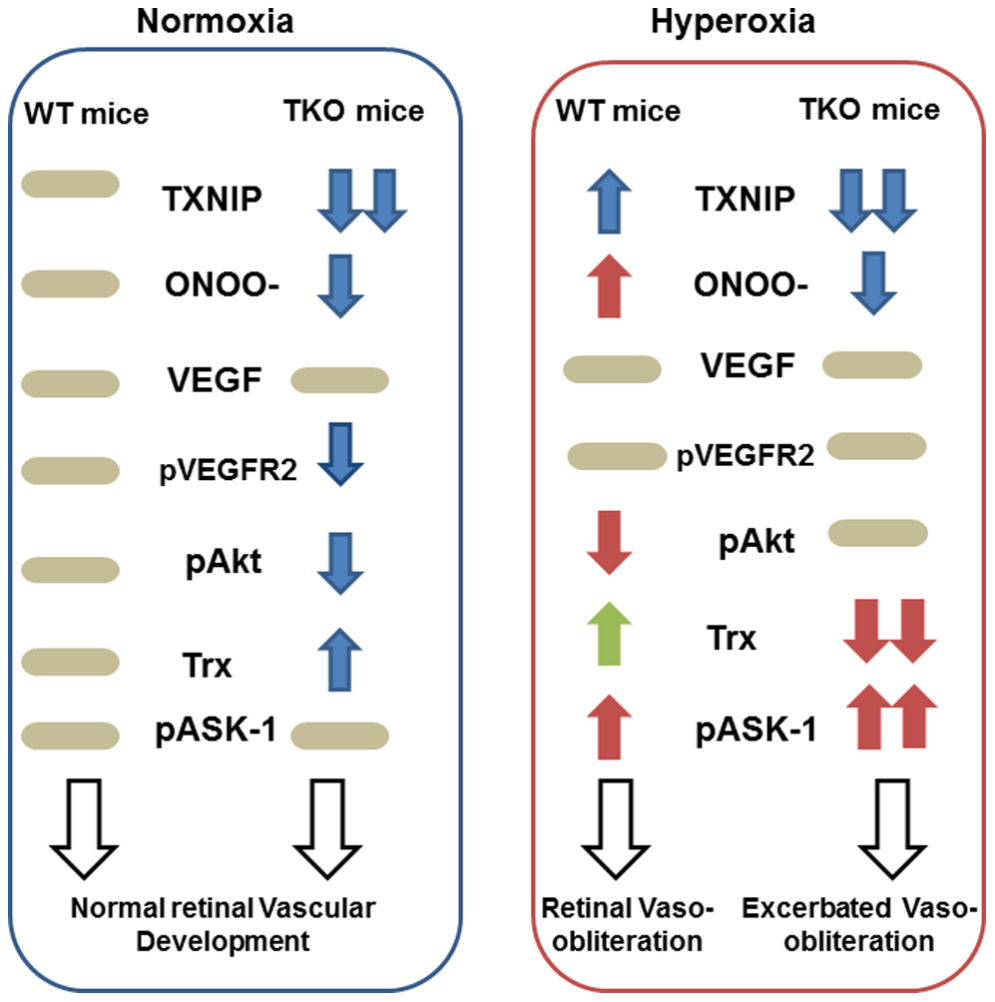
Representative diagram shows the impact of TXNIP deletion on retina vasculature under both normoxia and hyperoxia. Under normoxia, retinas from TXNIP-deficient mice showed similar VEGF levels, less peroxynitrite (ONOO-) levels, less VEGF receptor-2 (pVEGFR2) activation and upregulated thioredoxin (Trx) that collectively lead to normal vascular development in comparison to WT mice. Under hyperoxia, retinas from WT mice showed higher peroxynitrite formation, less survival Akt activation (pAkt) and upregulated proapoptotic signal of ASK-1 resulting in vaso-obliteration. Retinas from TKO although showed less peroxynitrite levels and maintained Akt activation, retinas experienced significant decreases in thioredoxin (Trx) that shift the balance of the ASK-1-Trx inhibitory complex and increases the activation of the proapoptotic ASK-1 pathway leading to exacerbated vasoobliteration compared to WT.

The main finding of the present study is: Despite increased antioxidant defense and decreased nitrative stress, TKO mice were more vulnerable to hyperoxia and had aggravated retinal vascular cell death (Fig. 1,2,5). These effects were not associated with changes in either retinal VEGF expression or activation of VEGFR-2/Akt (Fig. 3,4). Hyperoxia caused significant decrease in thioredoxin expression that was associated with activation ASK-1 apoptotic signal in TKO mice compared to WT (Fig. 6,7). These findings suggest that TXNIP expression is required for homeostasis of anti-apoptotic function of thioredoxin-ASK-1 complex in the retina in response to hyperoxia as depicted in Fig. 8.

Our current and previous work [23] demonstrated that retinas from TKO mice show similar level of vascular density at p7 and p12 to their WT-littermates, respectively. At normoxia, retinas from TKO have similar VEGF to WT mice, however higher level of Trx and less VEGFR2 activation suggesting compensatory mechanism of different angiogenic pathways (Trx-mediated pathway versus VEGFR2) that eventually resulted in normal retina vascular development. Nevertheless, exposure of TKO to drastic changes in oxygen level demonstrated different response of TKO in comparison to WT. Exposure of TKO to relative hypoxia further decreased VEGFR2 activation and angiogenic response [23]. Here, exposure of TKO to hyperoxia aggravated vascular cell death despite increasing activation of VEGFR2 to a comparable level of WT, suggesting differential response of TKO and activation of different signaling pathways in response to hyperoxia versus hypoxia. Oxygen toxicity has been attributed to increases in oxidative and nitrative stress that can modulate levels of VEGF, the main survival factor of endothelial cells [33] or activation of VEGFR2 and its downstream signal [27], [34]. We and others have demonstrated that preventing peroxynitrite formation and tyrosine nitration prevent capillary dropout in vivo [27], [29]-[31] and retinal endothelial cell death in vitro [34]-[36]. Indeed, exposure of WT mice to hyperoxia induced capillary dropout and tyrosine nitration that was associated with decreases in phosphorylation of the survival protein Akt. These results confirmed our previous findings using same model where WT retinas showed similar VEGF and VEGFR2 activation yet, impaired Akt activation due to nitration of p85, the regulatory subunit of the PI3-kinase [27], [34]. In comparison to WT, retinas from TKO mice demonstrated similar VEGF level and minimal levels of tyrosine nitration and less VEGFR2/Akt activation in normoxia as previously characterized [23]. However, upon exposure to hyperoxia, retinas from TKO showed improved activation of VEGFR2 and Akt that became comparable to WT but still TKP showed greater capillary dropout area. These findings suggest that decreases in nitration and restoration of Akt survival signal were not enough to rescue TKO from oxygen toxicity.

Several studies showed that TXNIP is a regulator of cellular redox status and has emerged as a key component in the link between redox regulation and the pathogenesis of diseases (reviewed in [1]–[3]). While targeting TXNIP represents an attractive strategy toward achieving less oxidative stress, and inflammation and preventing neurotoxicity [17], [37], [38], its genetic deletion provides different insight. Silencing or reducing TXNIP expression should result in increase in Trx availability with subsequent beneficial action. Indeed, retinas from TKO showed higher Trx at the mRNA and protein expression that resulted in significant increases in reduced-glutathione and thioredoxin-reductase activity at normoxia as reported previously [8], [23], [24]. Nevertheless, upon exposure to hyperoxia and the highly oxidative milieu, expression of Trx-1, Trx-2 mRNA and protein expression was enhanced in WT but decreased in TKO resulting in activation of cell death signal. The decrease in Trx was consistent with the transcriptional and total protein levels for both cytoplasmic (Trx-1) and mitochondrial (Trx-2) levels. In agreement, previous reports demonstrated that exposure to hyperoxia upregulated the protective Trx1 in alveolar epithelial cells and brain of developing rats [39], [40]. We believe that the TXNIP expression is important to maintain homeostasis of thioredoxin as anti-oxidant and anti-apoptotic and that genetic deletion of TXNIP will disturb cellular redox state. The finding that Trx compartmentalization under nitrosative/oxidative stress conditions is dependent on the expression levels of TXNIP [41] supports our hypothesis that TXNIP expression is important for Trx anti-apoptotic activity. Moreover, exposure to hyperoxia/oxidative stress can induce oxidation and inactivation of Trx-1 resulting in cell death as demonstrated in a lung model [42] and in a cellular model [43]. Recent studies by the same groups showed that shifting the antioxidant defense from thioredoxin system to glutathione is more beneficial in response to hyperoxia [44] and that glutathione/glutaredoxin system is essential for Trx reactivation after oxidation [43]. Of note, retinas from TKO experienced significant drop in reduced-GSH levels upon exposure to hyperoxia much more dramatic than WT mice (. 5.A)

We and others showed that Trx is not only an antioxidant, but it can also regulate cell survival by binding to and inhibiting the activity of apoptosis signal-regulating kinase 1 (ASK-1), a member of the MAP kinase kinase kinase family [14], [16], [45]. Trx can directly bind and inhibit ASK-1 or indirectly through inhibition of murine protein serine-threonine kinase 38 (MPK38), a member of the AMP-activated protein kinase-related serine/threonine kinase family that plays an important role in inducing ASK1-, TGF-β-, and p53-mediated apoptosis [45]. Here, we reported that hyperoxia caused reduction in Trx expression that was accompanied with significant increase in the activation of ASK-1. Increased activation of ASK-1 aggravated vascular cell death as confirmed with the increased expression of cleaved-caspase-3 and cleaved-PARP in TKO compared to WT. Our results lend further support to previous studies that demonstrated critical role of ASK-1 activation in hyperoxia-mediated injury [46], [47].

In summary, we believe that TXNIP expression is essential for Trx system homeostasis. We and others showed that TXNIP is required for VEGF angiogenic signal via redox-dependent [23] and independent mechanisms [25]. Here, we provide new insights that TXNIP is indispensable for homeostasis of thioredoxin anti-apoptotic function in the developing retina. Genetic deletion of TXNIP, despite increased cellular antioxidant defense, significantly accelerated vascular cell death in response of hyperoxia. These findings highlight the importance of manipulating the antioxidant defense specially during the possible control of retinopathy of prematurity, a potentially blinding disorders that affect premature infants [48].

## Supporting Information

Figure S1 **TXNIP knockout mice have similar retinal vascular density comparable to WT.** P7 retinas of both Wild type (WT) and TXNIP knockout (TKO) mice were fixed and stained with GS-IB4 conjugate-isolectin to quantify retinal vascular density. Images were processed via Image J software to be skeletonized to quantify vascular density. Our results showed no significant deference between TKO and WT in retinal vascular density during development at p7 compared to WT. (n = 12).
(TIF)
