# Supplementary figures and images for: Deletion of Thioredoxin Interacting Protein (TXNIP) Augments Hyperoxia-Induced Vaso-Obliteration in a Mouse Model of Oxygen Induced-Retinopathy
Source: PLoS One. 2014 Oct 16;9(10):e110388. doi: 10.1371/journal.pone.0110388 (PMC4199686; doi:10.1371/journal.pone.0110388)

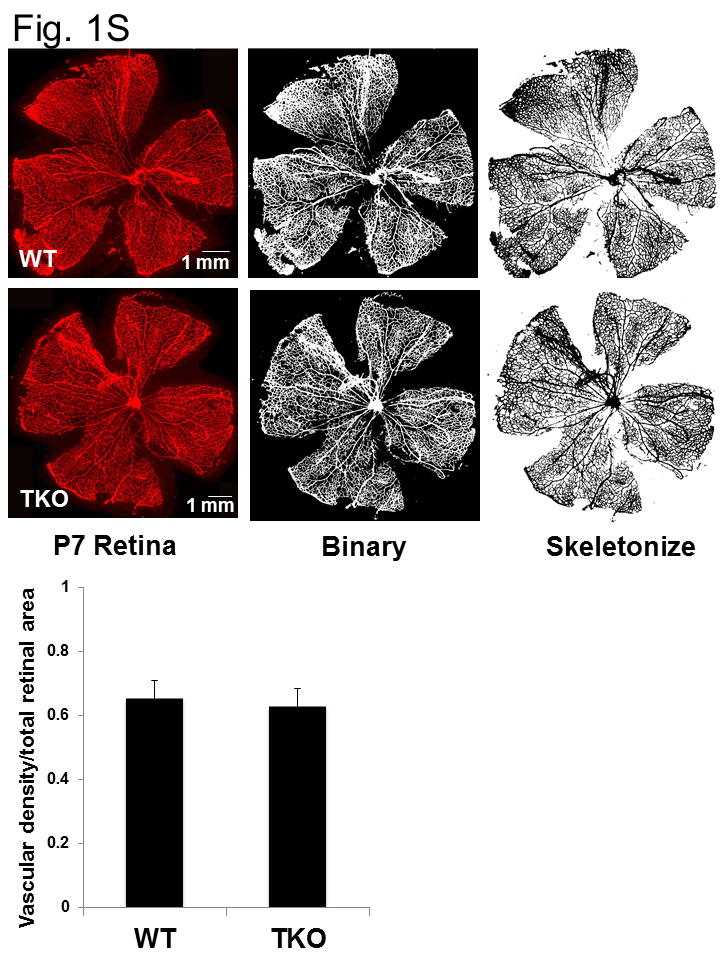

Supplement: Figure S1 — TXNIP knockout mice have similar retinal vascular density comparable to WT. P7 retinas of both Wild type (WT) and TXNIP knockout (TKO) mice were fixed and stained with GS-IB4 conjugate-isolectin to quantify retinal vascular density. Images were processed via Image J software to be skeletonized to quantify vascular density. Our results showed no significant deference between TKO and WT in retinal vascular density during development at p7 compared to WT. (n = 12). (TIF) [file pone.0110388.s001.tif]
